# Isolation, molecular characterization and phylogeny of *Naegleria* species in water bodies of North-Western Province, Sri Lanka

**DOI:** 10.1371/journal.pone.0248510

**Published:** 2021-03-11

**Authors:** Nuwan Gunarathna, Anjalie Amarasinghe, Sunil Wijesundara, Devika Iddawela, Susiji Wickramasinghe

**Affiliations:** 1 Faculty of Medicine, Department of Parasitology, University of Peradeniya, Peradeniya, Sri Lanka; 2 Ministry of Health, Ven. Baddegama Wimalawansa Thero Mawatha, Colombo, Sri Lanka; Manipal College of Medical Sciences, NEPAL

## Abstract

**Background:**

The inland freshwater bodies in the North-Western Province of Sri Lanka have ideal environmental conditions for the *Naegleria* species. Therefore, the presence and prevalence of *Naegleria* species in the water bodies of North-Western Province were determined by molecular characterization and phylogenetic analysis in this study.

**Methods:**

A total of 104 water bodies were selected from Kurunegala and Puttalam districts in the North-Western Province of Sri Lanka. Mean turbidity, pH, and temperature were recorded in each water body from three selected site. Centrifuged samples were cultured on non-nutrient agar plates with *Escherichia coli*. Enflagellation test positive isolates were subjected to DNA extraction and polymerase chain reaction using genus and species-specific primers targeting the internal transcribed spacer region (ITS) and *Mp2CL5* gene. Phylogenetic analysis was performed using Bayesian and maximum likelihood (ML) methods.

**Results:**

The prevalence of *Naegleria* species and *N*. *fowleri* in the study area were 23.07% and 1.92%. The prevalence of *Naegleria species* and the physicochemical parameters of the water bodies showed no significant correlation. Bayesian analysis of the ITS region revealed the *Naegleria* Sri Lankan (SL) isolates 1, 3, and 4 in a single clade separated from the 2 and 5. Furthermore, Bayesian analysis identified isolates 2 and 5 in the same clade with *Naegleria* sp. samples and *N*. *Philippinensis* forming a sister clade. However, in the ML tree, all isolates were in the same clade with *Naegleria* sp. samples and *N*. *Philippinensis*.

**Conclusions:**

The present study reports the first isolation of pathogenic *N*. *fowleri* from Sri Lanka. Based on Bayesian analysis, SL isolates 2 and 5 form a separate clade from 1, 3, and 4. However, in ML analysis, all isolates are grouped in one clade with *Naegleria* sp. samples and *N*. *philippinensis*. Further investigations are required to confirm these findings.

## Introduction

The genus *Naegleria* is a group of free-living amoebae (FLA) with more than 47 species [1]. *Naegleria fowleri* is the only species in this genus that is known to cause human disease. Trophozoites of *N*. *fowleri* can penetrate the cribriform plate and cause fulminant and primary amoebic meningoencephalitis (PAM) in humans. This disease is acquired while swimming, diving, or total submersion in water bodies contaminated with the infective stage of *N*. *fowleri* [2].

*Naegleria* species can be isolated from soil, air, and natural, industrial, and domestic water systems [3–5]. The global distribution of *Naegleria* species varies from 23% to 89%, depending on the geographic location [6]. Several studies have identified the prevalence of *Naegleria* spp. in Asia, including Thailand, China, and India [6,7–10].

Only two previous studies have reported the isolation of *Naegleria* species from Sri Lanka. However, *N*. *fowleri* is not reported in Sri Lanka [11,12]. These two previous studies have analyzed a limited number of samples taken from a few sampling sites. The present study covers the North-Western Province located in the dry zone of Sri Lanka, where there are a large number of water bodies that provide suitable environmental conditions for the optimum growth of *Naegleria* species. These water bodies are used frequently by the local people for their daily needs. Therefore, we investigated the presence of *Naegleria* species in the North-Western Province and analyzed their molecular characteristics. Furthermore, the present study reports the first isolation of *N*. *fowleri* from Sri Lanka.

## Materials and methods

### Study area

The present study was carried out in North-Western Province of Sri Lanka, located in the dry zone, and consisted of Kurunegala and Puttalam administrative districts. Approximately there are more than 6,000 water bodies in North-Western Province [13,14]. The total population of North-Western Province was approximately 2,370,075 with a land area of 7888 km^2^ [15], with 76 m of mean elevation above sea level and 2,316.1 mm of annual rainfall. Northeast monsoons in October and November provide the highest rainfall. The average annual temperature is 27.4°C, and the annual relative humidity is from 71% to 87% [16].

### Water sample collection

The sample size was decided by the formula given by Lachenbruch et al., 1991 [17]. The percentage of selecting a choice was 0.5, the confidence interval was 95%, and the calculated minimum sample number was 384. Water bodies in the North-Western Province were identified using 1:50,000 metric maps (1987) of the National Survey Department of Sri Lanka. As the map was not up to date over 20 years, satellite images were utilized to identify the water bodies (Map data@Google 2013). Filled, abandoned, and dried, water bodies were identified on the map. Abandoned or dried water bodies and water bodies situated in unreachable areas were excluded from the study. Only 104 filled water bodies were selected using a standard random number table. Accordingly, 64 and 46 water bodies were selected from Kurunegala and Puttalam districts.

Due to the seasonal monsoon rain in Sri Lanka, samples were collected for a long period, from April 2013 to September 2018. Each water body had several entryways. Based on animal and human contact, three different sites in a water body were selected for the sample collection. The first site was frequently used by local people, the second site was frequently used by local livestock, and the third site was minimally used by either humans or animals. Two water samples were collected from each site separately: one from the surface water and the other from deep water. Water samples were collected directly into sterile universal glass containers. The surface water samples were taken approximately 0.5 m away from the bank. The deep samples were taken approximately 1 m deep from the water level. Capped containers were dipped underwater then filled and were closed at the same deep level. Containers were labelled immediately after the collection, and the water samples were transported to the laboratory within 24 h of collection.

A total number of six water samples were collected (two samples from each of the three sites in one water body) from each water body to identify *Naegleria* spp. Altogether 624 samples were collected from 104 selected water bodies. Similarly, 624 water samples were collected from each site to analyze the physicochemical parameters in the field.

### Culture for free-living amoebae

Each sample was well-shaken and poured into 15 ml conical tubes. The tubes were centrifuged (2000 rpm for 2 min) and the sediments were used for inoculation. Cultures were inoculated on *Escherichia coli* (NCTC-10418) enriched agar plates as described by Ash and Orihel, (1987) [18]. The plates were sealed with parafilm and incubated overnight at 37°C. The sealed plates were examined on the following day under an inverted microscope (Leitz Diavert^®^). The examination of plates was continued for FLA up to 7 days before discarding.

### Enflagellation test

Scrapings from FLA positive culture plates were mixed with 1 ml of distilled water and incubated at 37°C for 30 min. Wet smears were prepared to observe enflagellation (transformation of trophozoites into pear-shaped bi-flagellates or multi flagellates) [19] under an inverted microscope *(*Leitz Diavert^®^). If enflagellation was not observed immediately, the solvents were incubated for 2 hours at 37°C [20], and samples were observed again before discarding.

#### *Naegleria* spp. identification by microscopic examination

*Naegleria* cysts and flagellates were harvested from culture plates positive for enflagellation test and examined by a light microscope (magnification x1000). The trichrome staining technique [21] was used in the morphological examination. *Naegleria* trophozoites, cysts, and flagellate forms were observed and the length of the trophozoites and flagellate and diameters of the cysts were measured [22].

### DNA extraction

The enflagellation test-positive culture plates were selected for DNA extraction. The surface of the agar plate was scraped with a sterile glass slide and washed thoroughly with 1.5 ml of distilled water. The washing was collected into a conical tube and centrifuged at 3000 rpm for 3 min. After that, 200 μl of the resulting deposit was taken for the DNA extraction using a PureLink^®^ Genomic DNA Kit according to the manufacturer’s guidelines.

### Polymerase chain reaction (PCR)

#### *Naegleria* genus-specific PCR

DNA of each extracted sample was amplified using two *Naegleria* genus-specific primers 1 and 2. The primers 1 and 2 amplified portions of the internal transcribed spacer region (ITS). The primer sequences, amplified regions, and amplicon sizes are given in Table 1. The polymerase chain reaction was performed in a thermal cycler (NYXTECHNIK, Amplytronix series, USA). Each reaction had 10x PCR buffer, 25 mM MgCl_2_, 2.5 mM deoxynucleotide triphosphate (dNTP), 10 pmol primers, and 0.5 μl Taq DNA polymerase (5 u/μl), a total volume of 25 μl. PCR conditions for primers 1 and 2 as follows; initial denaturation at 94°C for 3 min, 35 cycles of denaturation at 94°C for 30 s, annealing at 55°C for 30 s, extension at 72°C for 1 min and final extension step at 72°C for 5 min.

**Table 1:** Amplified regions and the primers used in the study.

**Primer number**	**Amplified region and size of the amplicon**	**Primer name and the sequence (5’-3’)**	**Reference**
		**F: Forward, R: Reverse**	
Primer 1	ITS region (311–500 bp)	F: (GAACCTGCGTAGGGATCATTT)	[23]
		R: (TTTCTTTTCCTCCCCTTATTA)	
		R: NFITSRW(AATAAAAGATTGACCATTTGAAA)	
Primer 2	ITS region (311–500 bp)	F: NGITS FW(AACCTGCGTAGGGATCATTT)	[24]
		R: NGITSRW(TTTCCTCCCCTTATTAATAT)	
		R: Y25(AAATGATCCCTACGCAGGTT)	
Primer 3	Mp2Cl5 gene		[25]
(**S**pecies specific nested PCR)	1^st^ PCR (166 bp)	F: Mp2Cl5 for (TCTAGAGATCCAACCAATGG)	
		R: Mp2Cl5 rev (ATTCTATTCACTCCACAATCC)	
	2^nd^ PCR (110 bp)	F: Mp2Cl5.for-in (GTACATTGTTTTTATTAATTTCC)	
		R: Mp2Cl5.rev-in (GTCTTTGTGAAAACATCACC)	

#### *Naegleria fowleri* species-specific PCR

DNA samples that were positive for *Naegleria* genus-specific primers were then subjected to PCR using nested PCR primers as shown in Table 1 (*N*. *fowleri* species-specific primers). These primers amplified the Mp2Cl5 membrane protein gene of *N*. *fowleri*.

Primer 3 was used for the nested PCR. Similar concentrations mentioned for the genus-specific PCR mixture were used, and the first PCR was carried out according to the conditions given in Sifuentes et al., 2014 [26]. The nested amplification was carried out using 5 μl of the first PCR product as the template. The conditions that were given for nested PCR in Réveiller, et al., 2002 and Marciano-Cabral et al., 2003 [25,27] were followed. Amplified products were analyzed by electrophoresis in 1.5% agarose gel and visualized under UV light.

### DNA sequencing, sequence annotation, and phylogenetic analysis

PCR products were purified (PureLink™ Genomic DNA Mini Kit) and sequenced using the PCR primers as sequencing primers in Applied Biosystem 3130 automated sequencer; Macrogen, Korea.

Sequences were manually edited using BioEdit v7.0.5.3 [28]. The sequence similarity search for each sequence was performed with the NCBI BLAST. Multiple sequence alignments with the reference sequences of *Naegleria* spp. available in the NCBI GenBank were obtained using ClustalW v2.0. Default gap penalties were given.

Bayesian inference was carried out in MrBayes [29] for ITS2 sequences, with the model as GTR+I+G. The frequency of clades in each tree was sampled for every 100 generations. Four Markov Chain Monte Carlo (MCMC) chains were run for one million generations as three heated chains and one cold chain. The four chains reached burn-in time by 250,000 generations. The maximum likelihood (ML) tree was constructed in MEGA v.7.0.26 [30].

Only one sequence was available for the Mp2CL5 gene in the GenBank. Therefore, a phylogenetic tree was not constructed for the *N*. *fowleri* sequences, and only the sequence alignment was carried out using MEGA v.7.0.26.

### Physicochemical analysis of the water samples

A portable digital turbidity meter (WGZ-1B), pH meter (Yuelong), and thermometer (TP300) were used to measure the physicochemical parameters in the water. The mean turbidity in Nephelometric Turbidity Units (NTU), pH, and water temperature (°C) of each sampling site were measured and recorded at the same time of sample collection.

### Statistical analysis

MINITAB (v.17) [31] software was used for the statistical analysis of physicochemical parameters. The recorded mean temperature, pH, and turbidity values of each sample were utilized for statistical analysis. Further, free-living amoeba, enflagellation test, and PCR positive samples in each sample site were subjected to statistical analysis.

## Results

### FLA detection by culture and enflagellation test

Of the total 624 samples, 320 (51.28%) were tested positive for FLA by culture (Table 2). Out of these, 123 (38.43%) were tested positive for the enflagellation test. Out of enflagellation positive samples, 108 (87.80%) were positive within the first hour, while the 15 (12.19%) samples took 1 to 3 hours for the transformation.

**Table 2:** Detected free living amoebae according to the sampling sites.

Sample site*	Number of culture plates with positive growth	Percentage (%) of culture plates with positive growth	Number of Positive enflagellation test
1S	59	18.44	22 (17.87%)
1D	50	15.63	18 (14.63%)
2S	64	20.00	29 (23.58%)
2D	47	14.69	16 (13.01%)
3S	55	17.18	25 (20.32%)
3D	45	14.06	13 (10.57%)
Total	320	100	123

*Samples are given a code indicating the sampling site (1^st^, 2^nd^, or 3^rd^) and the water level (S: Surface, D: Deep). E.g. 1S means the sample was from site 1 surface water.

### Morphological identification of *Naegleria* spp. by microscopic examination

**Fig 1:**
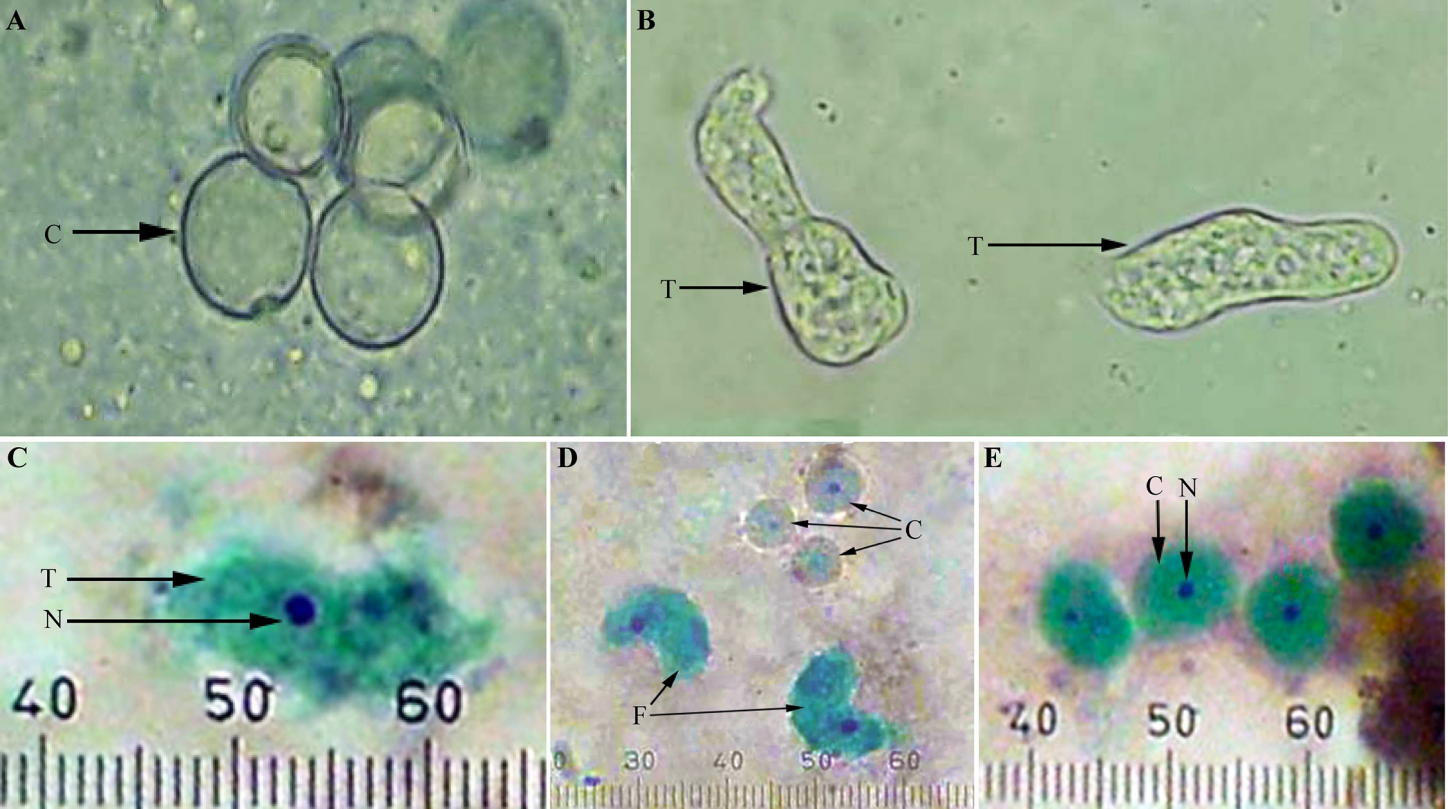
*Naegleria* cultures examined under the microscope. **A**: *Naegleria* cysts (without staining), arrow shows a cyst (C). **B**: *Naegleria* trophozoites (without staining), arrow shows a trophozoite (T)**. C**: *Naegleria* trophozoite (T) and the nucleus (N) in trichrome staining. **D**: *Naegleria* flagellates (F) and cysts (C) in trichrome staining. **E**: *Naegleria* cysts in trichrome staining show a cyst (C) and its nucleus (N).

The amoebae, cysts, and flagellate forms were identified according to the morphological features given in Cheesbrough (1981) [22] (Fig 1A and 1B). The trophozoites were active and constantly changed their size and shape. They moved by producing lobopodia, which were initially clear, but rapidly filled with granular cytoplasm (Fig 1B). The trophozoites showed a characteristic pattern of locomotion (a limax pattern).

The trichrome staining visualized nuclei in red and the cytoplasm in green colour. The length of the trophozoites was approximately 20–22 μm (Fig 1C). The cell wall of the cyst was smooth and single-layered. Harvested flagellates after 6 hours of the enflagellation test were 10–12 μm in length (Fig 1D). Cysts were round in shape and 7–9 μm in diameter (Fig 1E). Bi-flagellate forms demonstrated a limax pattern of locomotion.

### *Naegleria* spp. identification by PCR amplification and DNA sequencing

**Fig 2:**
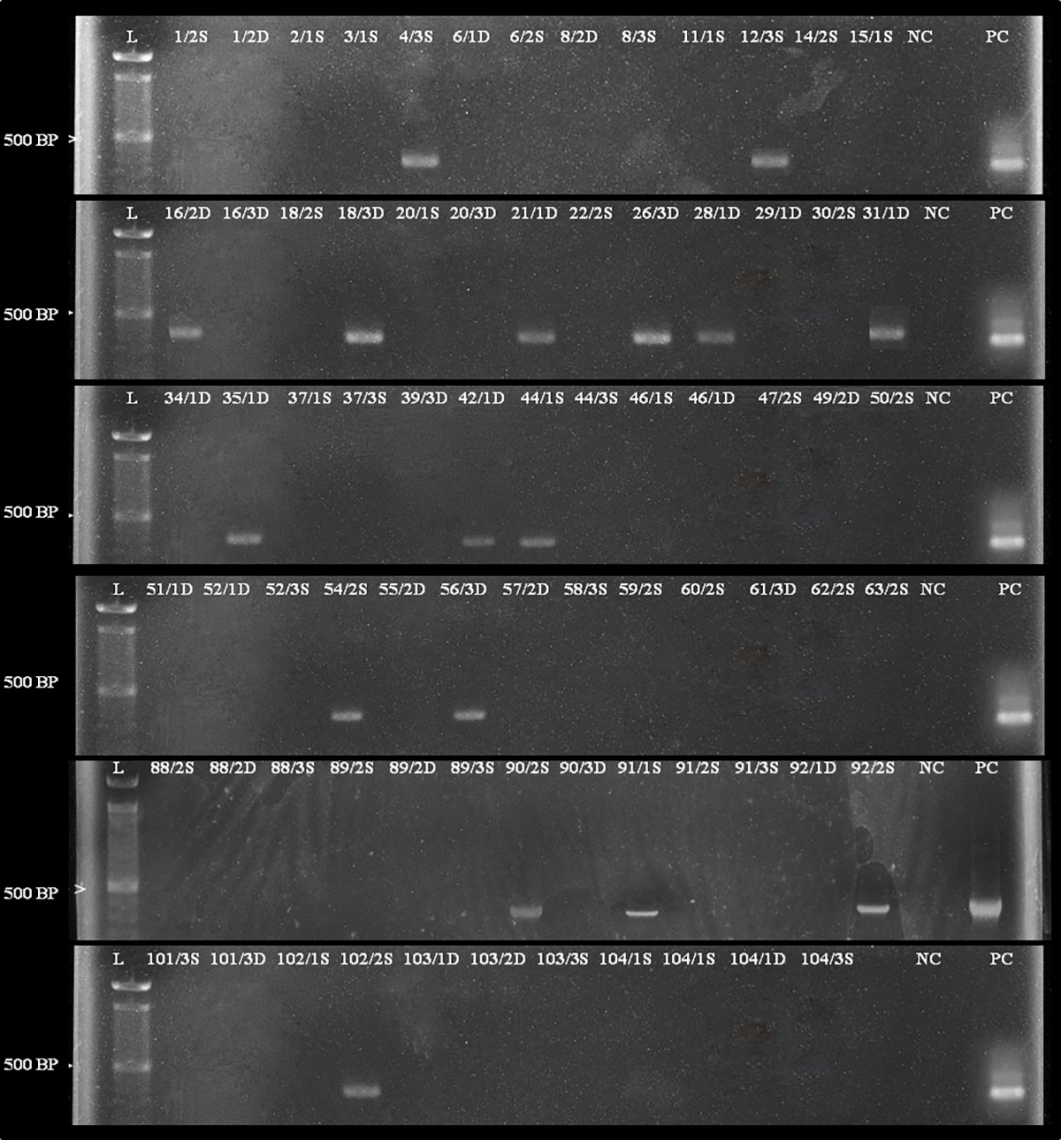
Amplified products of internal transcribed spacer (ITS) region for *Neagleria* species using genus-specific primer 1. L: 100 bp ladder; NC: Negative control; PC: Positive control; each sample is labelled by incorporating location, site, and depth of the collected sample. Among these samples 4/3S, 12/3S, 16/2D, 18/3D, 21/1D, 26/3D, 28/1D, 31/1D, 35/1D, 42/1D, 44/1S, 54/2S, 56/3D, 90/2S, 91/1S, 92/2S, and 102/1S show bands in the region of 500 bp.

Out of the 123 enflagellation positive samples, 17 were positive for primer 1 (Fig 2) and 7 were positive for primer 2 (S1 Fig). Five DNA sequences were obtained for primer 1. Nested PCR with primer 3 gave positive results for two samples (S2 Fig). The sequences resulted from this study are available in the GenBank (Acc. no. MN244451, MN244452, MN244453, MN244454, MN244455, MN475971, and MN475972).

### Sequence annotation and phylogenetic analysis

#### ITS region

**Fig 3:**
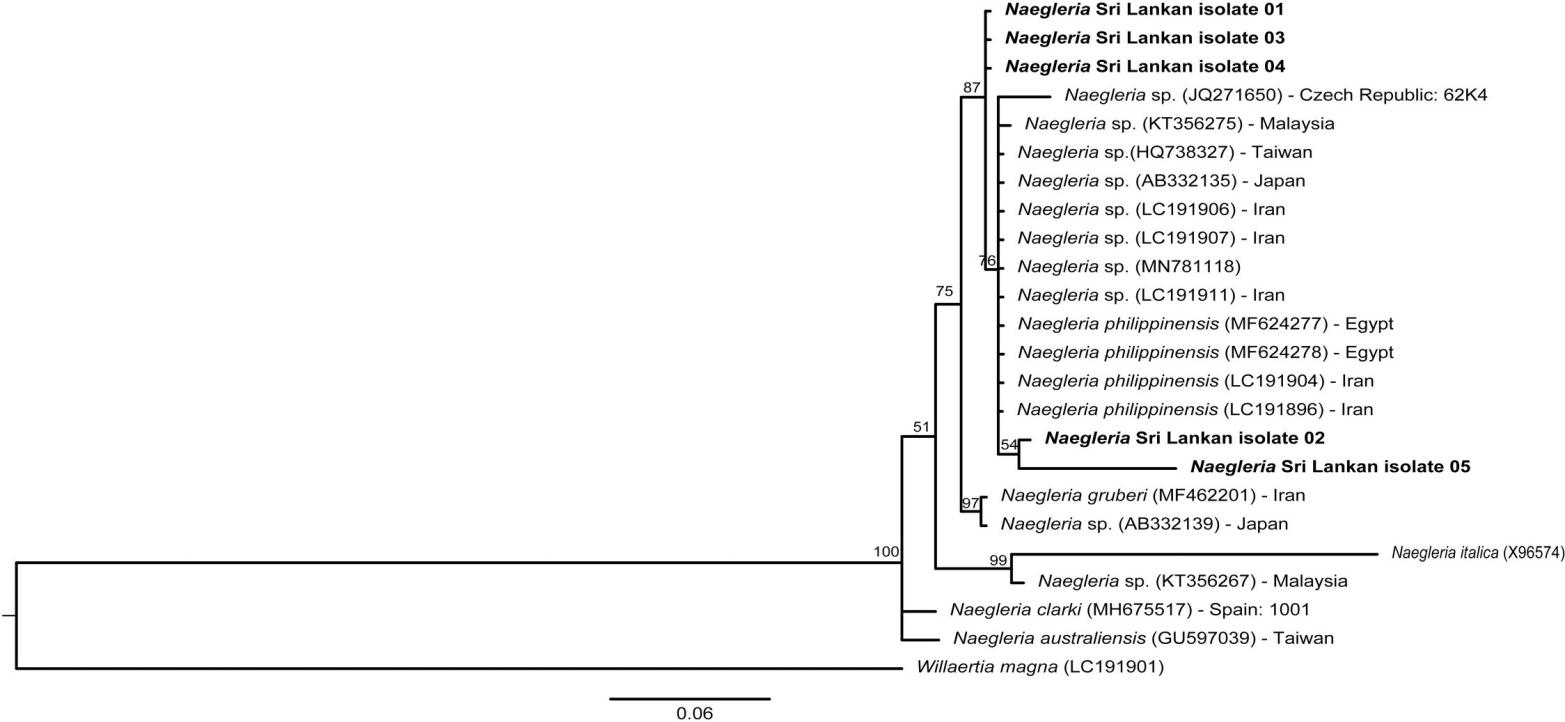
The Bayesian tree of *Naegleria* isolates for the ITS sequences. Accession numbers are indicated in parentheses. Isolated country and the availability of the strain are given. *Willaertia magna* (LC 191901) was used as the out-group. Scale bar represents 0.06 nucleotide divergence. Numbers above the nodes are the number of Bayesian posterior probability values (>70%).

Bayesian tree constructed with 24 taxa is shown in Fig 3. The BLAST search revealed that the isolates 1, 3, and 4 have 99.11% similarity with *Naegleria* sp. (GenBank acc. no. MN781118, LC191911, LC 191907, LC 191906) and 99.11% similarity with *N*. *philippinensis* (GenBank acc. no. LC191904, LC191896).

Isolate 2 showed 98.81% similarity with *Naegleria* species (GenBank acc. no. MN781188, LC191911, LC191907, LC191906) and 98.81% similarity with *N*. *philippinensis* (GenBank acc. no. LC191904, LC191896).

Isolate 5 showed 98.69% similarity with *Naegleria* species (GenBank acc. no.LC191906, LC191907, LC191911, LC191896, MN781118, MF624278, MF624277) and 98.69% similarity with *N*. *philippinensis* (GenBank acc. no. LC191904, LC191896).

Bayesian and ML analysis of the ITS region revealed the *Naegleria* Sri Lankan (SL) isolates 1, 3, and 4 in a single clade separated from the *Naegleria* SL isolates 2 and 5. Furthermore, Bayesian analysis identified isolates 2 and 5 in the same clade with *Naegleria* sp. samples and *N*. *Philippinensis* forming a sister clade (Fig 3). However, in the ML tree (S3 Fig), isolates 1, 3, and 4 were in the same clade with *Naegleria* sp. samples and *N*. *Philippinensis*.

#### Mp2CL5 gene

The *Naegleria* SL isolates 7 and 8 obtained for the Mp2CL5 gene sequence showed 100% similarity to *N*. *fowleri* (GenBank acc. no. AY049749) in the BLAST search and the sequence alignment.

### Prevalence of *N*. *fowleri* and other *Naegleria* species

The prevalence of *Naegleria* species in the surface water and deep water were 4.48% and 3.20% respectively. The total prevalence of *Naegleria* spp. in the study area was 23.07%. The *N*. *fowleri* was detected in Periyakulama *wewa* (Puttalam District) and Dignnewa *wewa* (Kurunegala District). The prevalence of *N*. *fowleri* was 1.92% in the study area.

### Physicochemical analysis of the water samples

The temperature, pH, and turbidity of water samples varied within 25–39°C, 7.10–6.82 pH, and 44–723 NTU respectively (S1 Table). Temperature and the pH of surface water samples were significantly higher than that of deep-water samples (P<0.05) (S1 Table). The mean turbidity was significantly high in deep water than surface water samples (P<0.05) in all three sample sites (1^st^, 2^nd^, and 3^rd^). The mean temperature and pH of surface and deep water samples of the 1^st^ site were significantly higher than the 2^nd^ and 3^rd^ site samples (P<0.05).

## Discussion

This study focus on the detection and identification of *Naegleria* species present in the dry zone of North-Western Province. In the present study, the amoebae, cysts, and flagellate forms were observed, and the bi-flagellates were observed with a typical locomotion pattern. Among 123 enflagellation test positive samples, only 24 were positive for *Naegleria* by PCR. This difference could be due to false-positive results of the enflagellation test. A previous study confirms the nonspecificity of enflagellation test in the identification of the genus *Naegleria* [32].

The global prevalence of *Naegleria* species varies from 28% to 89% [33]. The overall prevalence of *Naegleria* species in the present study area is 23.07%. A similar prevalence rate to the *Naegleria* species in the present study was reported in Egyptian aquatic environments (24.6%) [34]. In contrast, a lower prevalence of *Naegleria* species was reported in a study conducted in Taiwan (4%) [7].

The prevalence of *N*. *fowleri* identified in two water bodies in Kurunegla and Puttalam districts in this study is 1.92%. A meta-analysis conducted in 2020 identified the global prevalence of *N*. *fowleri* as 23.27% [35]. There are indications that climate change tends to increase the abundance and range of *N*. *fowleri* [36]. From 1965 to 2016, there were 381 PAM cases identified globally, and the number of reported cases increased at a rate of 1.6% per year [37]. To date, neither PAM nor *N*. *fowleri* is reported in Sri Lanka. However, according to the Sri Lankan Annual Health Bulletin of 2016 [38], approximately 200 deaths due to encephalitis of unknown etiology from 1996 to 2015 were reported in the country.

*Naegleria* species isolated from this study did not significantly confine to any specific sampling site (p>0.05). We identified a higher prevalence of *Naegleria* in surface water (4.48%) than in the deep-water samples (3.20%). However, there was no statistical significance in the reported differences (p>0.05). A previous study conducted in Sri Lanka identified *Naegleria* thrive more significantly in surface water than in deep-water [39]. Two different studies conducted in the United States of America recorded a higher prevalence rate of *Naegleria* in surface water [40,41]. A higher prevalence of *Naegleria* in surface water, compared to the present study, was reported in India (34.5%), Iran (15%), and Japan (68.7%) [6,42,43]. Previous studies identified the prevalence rate *of Naegleria* species in deep water [44–46]. *N*. *fowleri* was also isolated in deep wells [47], the groundwater system, and geothermal water [48]. Moreover, deep water diving has caused PAM [49].

The phylogenetic analysis carried out by Bayesian and ML methods for the ITS region revealed a *Naegleria* clade containing all SL isolates and *N*. *philippinensis* (Fig 3). The Bayesian method identified SL isolates 1, 3 and, 4 in a sister clade to the *N*. *philippinensis* clade, separated from SL isolates 2 and 5. The ML analysis gave a single clade with all the SL isolates and *N*. *philippinensis* reference sequences. Sequence analysis identified more than 98% similarity between SL isolates and *N*. *philippinensis* sequences. Therefore, according to the sequence analysis and phylogenetic relationships identified in this study, we can suggest that SL isolates could be *N*. *philippinensis*, but they can be different stains. However, in the present study, the occurrences of *Naegleria* species were not significantly associated with the assessed physicochemical parameters of the sampling sites.

## Conclusions

The pathogenic *N*. *fowleri* was identified in Sri Lanka for the first time in this study. Furthermore, based on Bayesian analysis of *Naegleria* SL isolates 1, 3, and 4 in a single clade separated from the *Naegleria* SL isolates 2 and 5. However, in the ML tree isolates 1, 3, and 4 were in the same clade with *Naegleria* sp. samples and *N*. *Philippinensis*. Therefore, further investigations should be carried out to confirm the species of *Naegleria* found in this study. The presence of the *N*. *fowleri* in the country indicates the necessity for elaborate studies extended to identify *Naegleria* species in different water sources in other parts of Sri Lanka.

## Supporting information

S1 Fig Amplified products of internal transcribed spacer (ITS) region for *Neagleria* species using-genus specific primer 2. L: 100 bp ladder; NC: Negative control; PC: Positive control; each sample is labelled by incorporating location, site, and depth of the collected sample. Among these samples 64/2S, 67/2S, 73/2S, 77/1D, 78/3S, 85/1S, and 87/2S show bands in the region of 500 bp.
(TIF)

S2 Fig Nested PCR products of Mp2Cl5 membrane protein gene of *N*. *fowleri* using *N*. *fowleri* species-specific primer. Lanes L: 100 bp ladder; NC: Negative control; PC: Positive control; each sample is labelled by incorporating location, site, and depth of the collected sample. Among these samples, 77/1D and 78/3S show positive results.
(TIF)

S3 Fig Maximum likelihood (ML) tree obtained by the MEGA v.7.0.26. Numbers above the nodes indicate bootstrap values (>50%). Scale bar represents 0.05 nucleotide divergence. *Naegleria* SL isolates 1, 3, and 4 are in the same clade with *N*. *philippinensis*. *Naegleria* SL isolates 2 and 4 are in a separate clade from SL isolates 1, 3, and 4.
(TIF)

S1 Table GIS readings, temperature, pH, and turbidity values of *Naegleria* PCR positive samples. (TIF)
