# Supplementary figures and images for: Isolation, molecular characterization and phylogeny of Naegleria species in water bodies of North-Western Province, Sri Lanka
Source: PLoS One. 2021 Mar 11;16(3):e0248510. doi: 10.1371/journal.pone.0248510 (PMC7951808; doi:10.1371/journal.pone.0248510)

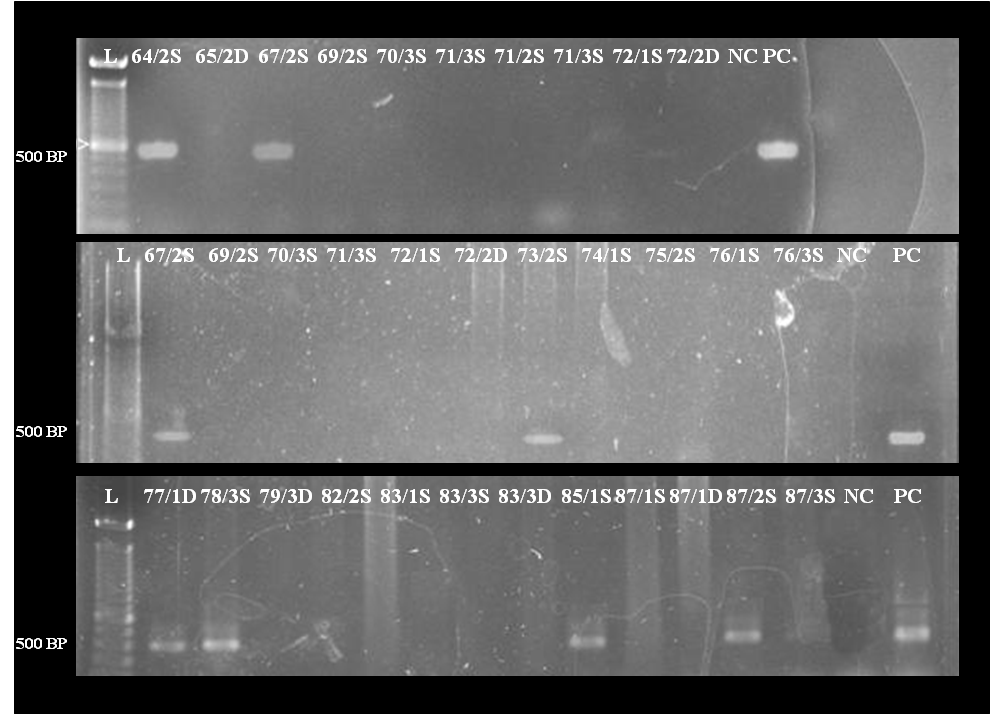

Supplement: S1 Fig — L: 100 bp ladder; NC: Negative control; PC: Positive control; each sample is labelled by incorporating location, site, and depth of the collected sample. Among these samples 64/2S, 67/2S, 73/2S, 77/1D, 78/3S, 85/1S, and 87/2S show bands in the region of 500 bp. (TIF) [file pone.0248510.s001.tif]

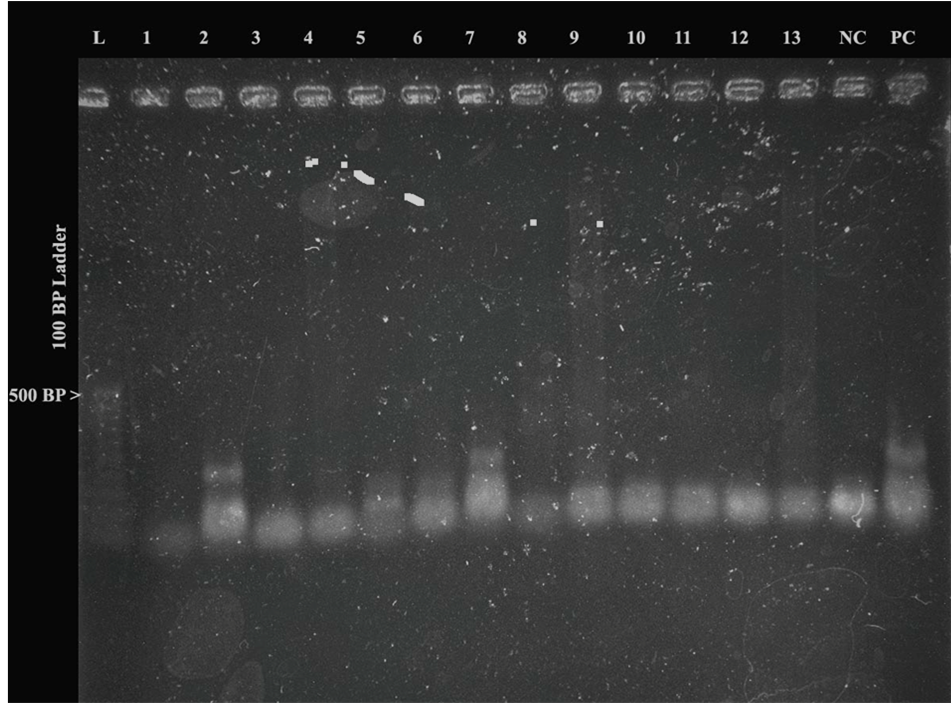

Supplement: S2 Fig — Lanes L: 100 bp ladder; NC: Negative control; PC: Positive control; each sample is labelled by incorporating location, site, and depth of the collected sample. Among these samples, 77/1D and 78/3S show positive results. (TIF) [file pone.0248510.s002.tif]

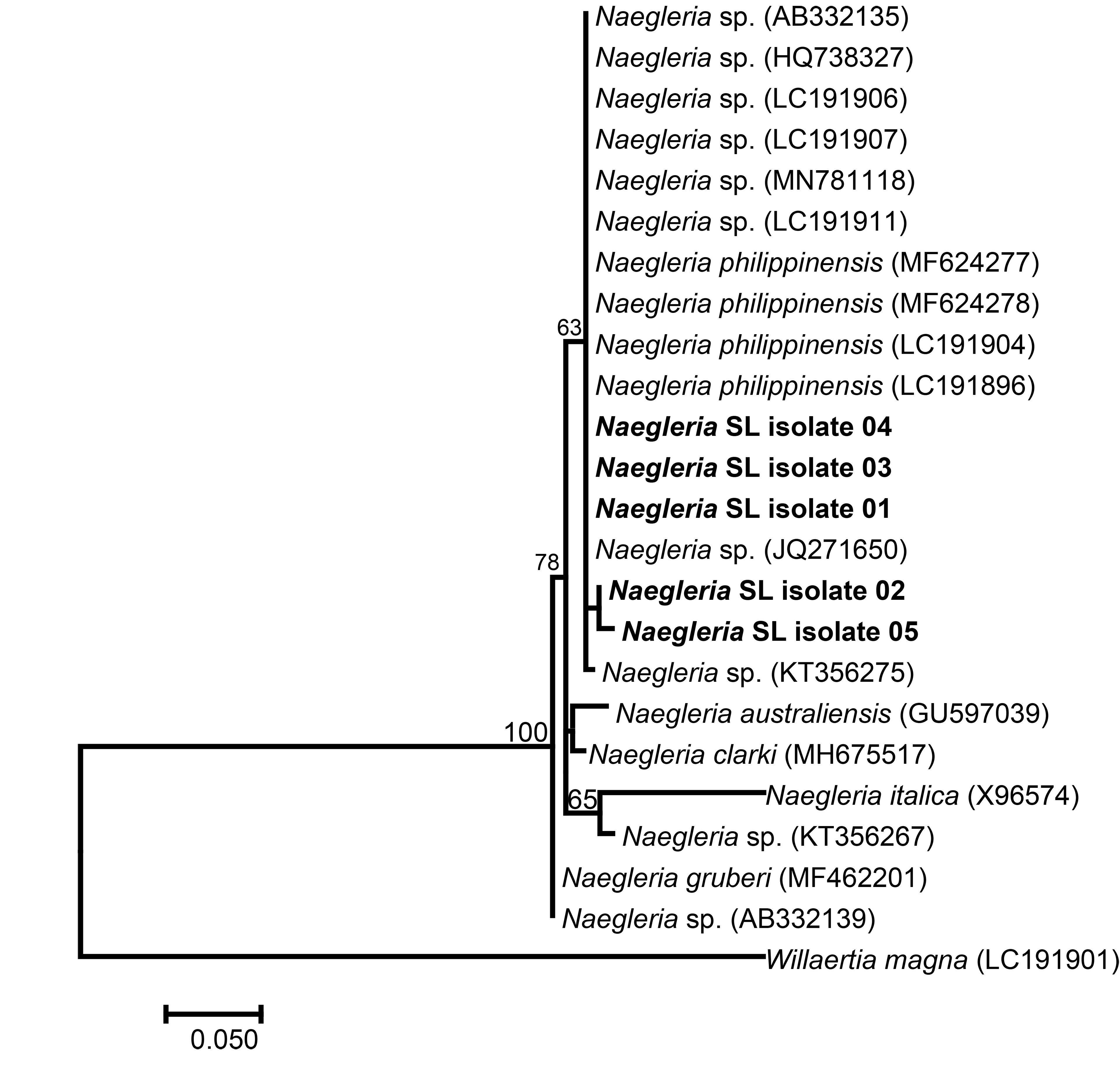

Supplement: S3 Fig — Numbers above the nodes indicate bootstrap values (>50%). Scale bar represents 0.05 nucleotide divergence. Naegleria SL isolates 1, 3, and 4 are in the same clade with N. philippinensis. Naegleria SL isolates 2 and 4 are in a separate clade from SL isolates 1, 3, and 4. (TIF) [file pone.0248510.s003.tif]

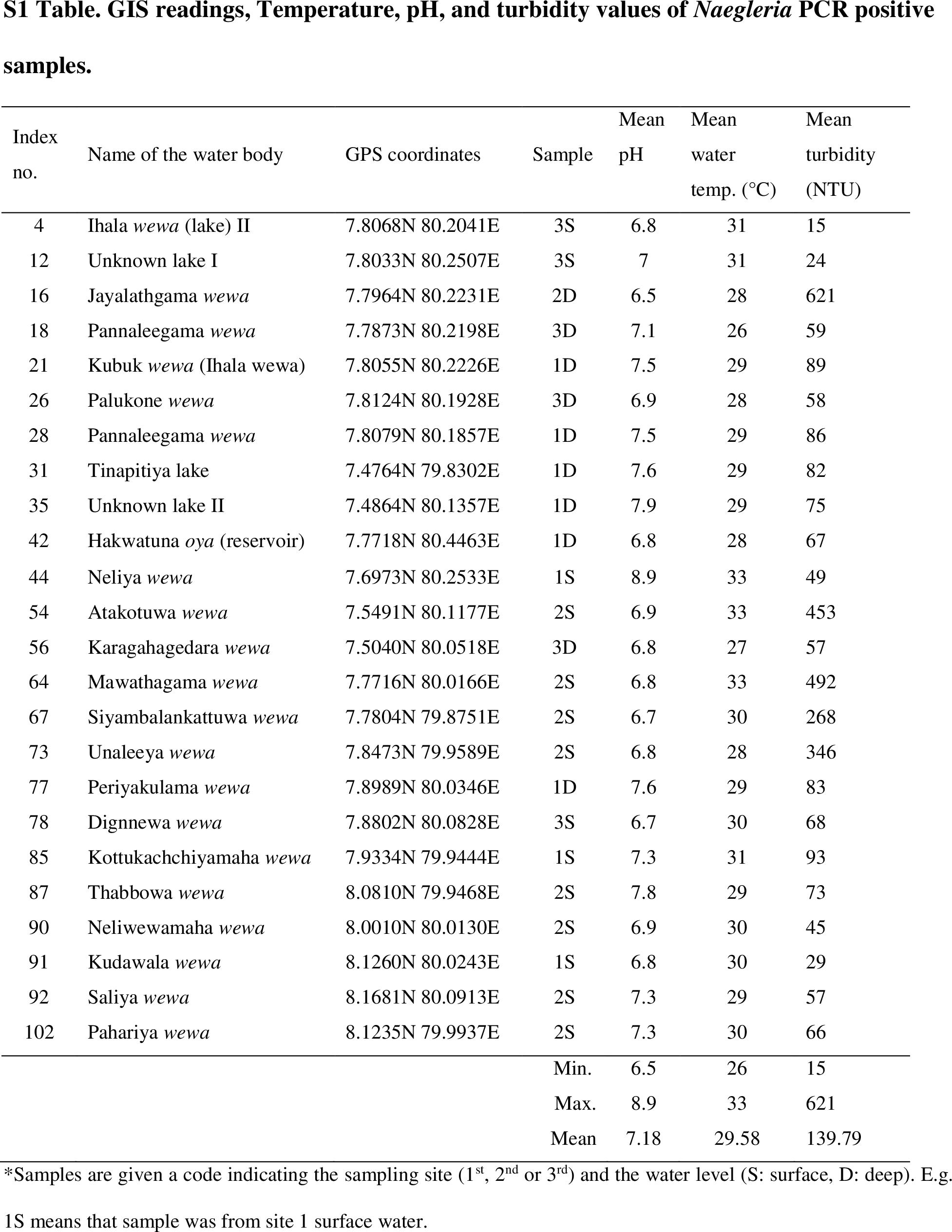

Supplement: S1 Table — (TIF) [file pone.0248510.s004.tif]
